# YqeH contributes to avian pathogenic *Escherichia coli* pathogenicity by regulating motility, biofilm formation, and virulence

**DOI:** 10.1186/s13567-022-01049-6

**Published:** 2022-04-18

**Authors:** Lei Yin, Baoyan Cheng, Jian Tu, Ying Shao, Xiangjun Song, Xiaocheng Pan, Kezong Qi

**Affiliations:** 1 Livestock and Poultry Epidemic Diseases Research Center of Anhui Province, Institute of Animal Husbandry and Veterinary Science, Anhui Academy of Agricultural Sciences, Hefei, 230031 Anhui China; 2 Anhui Province Key Laboratory of Veterinary Pathobiology and Disease Control, College of Animal Science and Technology, Anhui Agricultural University, Hefei, 230036 China

**Keywords:** Avian pathogenic *Escherichia coli*, *yqeH*, motility, biofilm formation, virulence, pathogenicity

## Abstract

Avian pathogenic *Escherichia coli* (APEC) is a pathotype of extraintestinal pathogenic *E. coli* and one of the most serious infectious diseases of poultry. It not only causes great economic losses to the poultry industry, but also poses a serious threat to public health worldwide. Here, we examined the role of YqeH, a transcriptional regulator located at *E. coli* type III secretion system 2 (ETT2), in APEC pathogenesis. To investigate the effects of YqeH on APEC phenotype and virulence, we constructed a *yqeH* deletion mutant (APEC40-Δ*yqeH*) and a complemented strain (APEC40-CΔ*yqeH*) of APEC40. Compared with the wild type (WT), the motility and biofilm formation of APEC40-Δ*yqeH* were significantly reduced. The *yqeH* mutant was highly attenuated in a chick infection model compared with WT, and showed severe defects in its adherence to and invasion of chicken embryo fibroblast DF-1 cells. However, the mechanisms underlying these phenomena were unclear. Therefore, we analyzed the transcriptional effects of the *yqeH* deletion to clarify the regulatory mechanisms of YqeH, and the role of YqeH in APEC virulence. The deletion of *yqeH* downregulated the transcript levels of several flagellum-, biofilm-, and virulence-related genes. Our results demonstrate that YqeH is involved in APEC pathogenesis, and the reduced virulence of APEC40-Δ*yqeH* may be related to its reduced motility and biofilm formation.

**Supplementary Information:**

The online version contains supplementary material available at 10.1186/s13567-022-01049-6.

## Introduction

Avian pathogenic *Escherichia coli* (APEC), a pathotype of extraintestinal pathogenic *E. coli* (ExPEC), causes serious infectious diseases in poultry [1]. Different APEC serotypes cause local or systemic infections in poultry, including respiratory infections, sepsis, polyserositis, coligranuloma, cellulitis, yolk sac infection, omphalitis, and swollen head syndrome, resulting in significant economic losses to the poultry industry [2]. Understanding the underlying molecular mechanisms of APEC pathogenicity is crucial for controlling avian colibacillosis.

There are many transcriptional regulators in Gram-negative pathogenic bacteria, which play important roles in regulating bacterial metabolism and the expression of virulence genes. More than 250 transcription factors are known to regulate gene expression in *E. coli*, and coordinate the expression of numerous promoters in response to specific environmental cues [3–5]. Although many virulence factors are known to be associated with APEC pathogenicity, the regulation of their expression is still not fully understood.

In *E. coli*, two distinct type III secretion systems (T3SSs) have been identified and characterized. The locus of enterocyte effacement (LEE), which encodes T3SS in intestinal *E*. *coli*, such as enteropathogenic *E*. *coli* and enterohemorrhagic *E*. *coli* (EHEC), is essential for the formation of attaching and effacing (A/E) lesions. A second T3SS, *E*. *coli* type III secretion system 2 (ETT2), plays an important role in the invasion, intracellular survival, and virulence of ExPEC, including APEC and newborn meningitis *E*. *coli* [6, 7]. We speculated that the transcriptional regulators located in the ETT2 cluster play an important role in APEC pathogenicity. Many reviews have described the various transcriptional regulator genes located in the ETT2 cluster, including *yqeK*/*etrB*, *etrA*, and *eivF*, which encode transcription factors that regulate the expression of virulence genes in intestinal pathogenic *E*. *coli* [8]. For example, EtrA and EivF of EHEC O157 negatively affect LEE expression and bacterium adherence to epithelial cells [9]. In contrast, the regulator YqeK/EtrB activates LEE expression and promotes A/E lesion formation by directly interacting with the Ler regulatory region [10]. Identifying new transcriptional regulators and their functions will clarify ETT2 pathogenesis.

In this study, we investigated the role of the transcriptional regulator YqeH located at ETT2 locus in the pathogenesis of APEC infections. The deletion of *yqeH* significantly reduced the virulence of APEC in vivo and in vitro. This was associated with reductions in the expression of many virulence-associated genes regulated by YqeH, which reduced bacterium motility, biofilm formation, adhesion, and invasion, suggesting that YqeH is involved in the pathogenicity of APEC.

## Materials and methods

### Bacterial strains, plasmids, and growth conditions

The strains and plasmids used in this study are listed in Table 1. The nucleotide sequences of the primers (GenBank: HG738867.1) are listed in Additional file 1. APEC strain APEC40 [11] (serotype O18:K1) of the B2 and ST95 phylogenetic groups, was isolated from a chicken with clinical septicemic symptoms of colibacillosis in Anhui, China. All *E. coli* strains were grown in Luria–Bertani (LB) medium at 37 °C with aeration. LB medium was supplemented with ampicillin (Amp, 100 mg/mL) and chloramphenicol (Cm, 30 mg/mL), unless specified otherwise.

**Table 1:** **Strains and plasmids used in this study**

Strains or plasmids	Description	Reference
Strains		
APEC40	wild-type strain, isolated from sick chicken	This study
APEC40-△*yqeH*	*yqeH* deletion mutant in APEC40	This study
APEC40-C△*yqeH*	APEC40-△*yqeH* with plasmid pSTV28-*yqeH*	This study
DH5a	F-,△*(lacZYA-argF)U169,recA1,endA1,hsdR17 (rk-,mk* +*), phoA,supE44,* γ*-*	TIANGEN
Plasmids		
pKD46	Amp; expresses γ red recombinase	This study
pKD3	Cm gene, template plasmid	This study
pCP20	Cm, Amp, yeast Flp recombinase gene, FLP	This study
pSTV28	Cm, lacZ	Takara
pSTV28-*yqeH*	pSTV28 derivative harboring *yqeH* gene	This study

### Ethics statement

The animal experiments were approved by the Institutional Animal Care and Use Committee at the Institute of Animal Husbandry and Veterinary Science, Anhui Academy of Agricultural Sciences (Permit No: AAAS-IAHVS-Po-2020-0034). The chickens were employed in experiments that complied with the ARRIVE guidelines of laboratory animal welfare and ethics [12].

### Mutant construction and complementation plasmids

A *yqeH* deletion mutant strain was generated with the lambda Red recombinase system, as described previously [13]. Linear PCR products were transformed into APEC40 carrying the *pKD*46 plasmid and the *yqeH* gene was replaced with a chloramphenicol resistance cassette. The chloramphenicol-resistance cassette was then eliminated by the helper plasmid pCP20 and a chloramphenicol-sensitive mutant strain was selected. The mutant strain was confirmed with PCR and DNA sequencing, and designated APEC40-Δ*yqeH*. To generate a complemented strain, the *yqeH* gene (including its putative promoter) was amplified and subcloned into plasmid pSTV28 with the primer pair *yqeH* Co-F and *yqeH* Co-R (Additional file 1). The mutant strain APEC40-Δ*yqeH* was then transformed with recombinant plasmid pSTV28-*yqeH* to generate the complemented strain APEC40-CΔ*yqeH*.

### Determination of bacterial growth, motility, and biofilm formation

Bacterial growth was measured as described previously [14]. Briefly, strains APEC40, APEC40-Δ*yqeH*, and APEC40-CΔ*yqeH* were cultured in LB medium until the optical density at 600 nm (OD_600_) reached 1.0. An equal amount of each bacterial culture was then transferred into 100 mL of LB medium in a volumetric ratio of 1:100 (v/v) and incubated at 37 °C with shaking. OD_600_ was monitored at 2 h intervals with a spectrophotometer (Bio-Rad, USA). The experiment was repeated three times and all samples were measured in triplicate.

The swarming motility of strains APEC40, APEC40-Δ*yqeH*, and APEC40-CΔ*yqeH* was assessed on soft-agar plates (0.25% agar), as previously described [15]. Briefly, bacteria were grown to an OD_600_ of 1.0 in LB medium and pelleted by centrifugation. The resulting pellet was washed and suspended in phosphate-buffered saline (PBS). The bacterial suspension (2 μL) was spread by point inoculation in the center of the soft-agar plates and incubated at 37 °C overnight. Swarming motility was assessed by measuring the diameter of migration. The experiment was repeated three times and all samples were measured in triplicate. In addition, the strains APEC40, APEC40-Δ*yqeH*, and APEC40-CΔ*yqeH* were negatively stained using 2% phosphotungstic acid (Sigma). Finally, the stained bacteria were deposited on a carbon-coated grid, followed by observation under a FEI T12 transmission electron microscope (FEI, Ltd, Hillsboro, OR, USA).

An assay involving crystal violet staining was used to quantify biofilm formation by APEC, as previously described [11]. Briefly, the strains were grown overnight in 5 mL of LB medium at 37 °C with rotation at 200 rpm. The cultures were suspended in M9 minimal medium supplemented with 0.2% fructose, and diluted to an OD_600_ of 0.1. An aliquot (200 μL) of each cell suspension was transferred to a 96-well plate (Corning, Corning, NY, USA) and incubated at 37 °C for 36 h. The wells were washed gently three times with PBS and stained with 0.1% crystal violet for 30 min at room temperature. The wells were then rinsed three times with PBS, air-dried, and 100 μL of 95% ethanol was added to dissolve the crystal violet. OD_595_ was measured with a Synergy 2 micro-plate reader (BioTek, USA).

### Bacterial adhesion and invasion assays

Bacterial adhesion and invasion assays were performed as described previously [16]. Chicken embryo fibroblast DF-1 cell monolayers were washed with Dulbecco’s modified Eagle’s medium (DMEM) without fetal bovine serum, and infected with bacteria at a multiplicity of infection (MOI) of 100 for 2 h at 37 °C under 5% CO_2_. After the cells were washed with PBS, they were lysed with 0.5% Triton X-100, and the bacteria were counted by plating them on LB agar plates. For the invasion assay, cell cultures were inoculated with bacteria as described for the bacterial adhesion assay. After incubation for 1 h, the cells were washed and treated with DMEM containing gentamicin (100 µg/mL) for 1 h to kill any extracellular bacteria. The monolayers were then washed and lysed with 0.5% Triton X-100. Serial dilutions of the cell suspensions were spread onto LB agar plates, and after overnight growth at 37 °C, the colony-forming units (CFUs) were counted. The input dilution of the bacteria was also plated to determine the CFU count for each inoculum.

### Animal infection experiments

The virulence of strains APEC40, APEC40-Δ*yqeH*, and APEC40-CΔ*yqeH* was determined in chicks. The APEC strains were grown to the exponential phase and collected, washed three times in PBS, and then adjusted to the appropriate concentration. For each group, eight 7-day-old Roman chicks were inoculated intratracheally with 10^8^ CFUs of bacteria (APEC40, APEC40-Δ*yqeH*, and APEC40-CΔ*yqeH*, respectively), or with PBS as negative control. Mortality was monitored daily up to 7 days post infection.

Bacterial colonization was determined during systemic infections, as described previously [17]. At 24 h post-infection, the chicks were bled, euthanized, and dissected. The bacterial loads in the blood were determined by plating the bacteria onto LB agar plates. The chick livers, spleens, and lungs were collected, weighed, and homogenized. The homogenates were diluted and plated onto LB agar to determine the bacterial numbers.

### RNA-Seq transcriptomic assay

The transcriptional levels of APEC40 and APEC40-Δ*yqeH* cells were determined, as described previously [18], with some modification. To evaluate the effects of YqeH on the transcriptional levels in APEC40 and APEC40-Δ*yqeH*, the cells were collected by centrifugation at 12 000 rpm for 5 min from cultures in LB medium when OD_600_ attained 0.80. The collected cells were washed with PBS (pH 7.4) and centrifuged. Total RNA was extracted from the cells with TRIzol Reagent (Invitrogen, Thermo Fisher Scientific, USA), according to the manufacturer’s instructions, and the genomic DNA was removed with DNase I (TaKaRa, Japan). cDNA was synthesized with the SuperScript™ Double-Stranded cDNA Synthesis Kit (Invitrogen, Thermo Fisher Scientific) and sequenced by Shanghai Major BioBiopharm Technology Co. Ltd. (Shanghai, China) with the Illumina HiSeq2500 System (Illumina, CA, USA). The differential expression analysis was performed with the EdgeR software. Differences in expression levels between groups were considered significant after adjustment was made for multiple testing, based on a q-value < 0.05. The genes were first filtered, based on q < 0.05 and an absolute difference > twofold, when |log_2_fold change|> 1.0. The enrichment of the differentially expressed genes was analyzed with Kyoto Encyclopedia of Genes and Genomes (KEGG) pathway analysis was performed with KOBAS.

### RNA isolation and reverse transcription–quantitative PCR (RT–qPCR)

RNA isolation and RT–qPCR (primers listed in Additional file 1) were performed as described previously [19]. The bacterial strains were grown to post-exponential phase (OD_600_ = 0.80) in the absence of antibiotics. Total RNA was isolated with the EasyPure^®^ RNA Kit (TransGen Biotech Co., Ltd, China), according to the manufacturer’s instructions. The quality of the RNA was determined, cDNA was synthesized, and a microarray analysis performed. SYBR Green detection was used for the RT–qPCR. Each reaction was conducted in triplicate in two-step multiplex qPCR assays with SYBR_®_ Premix Ex Taq™ (TransGen Biotech Co., Ltd), and bacterial *dnaE* was used as the internal reference gene. The relative expression levels were measured with the 2^−ΔΔCT^ method [19].

### Detection of transcription level of type 1 fimbriae genes by RT–qPCR

The transcription level of type 1 fimbriae genes (*fimA*, *fimB*, *fimC*, *fimD*, *fimE*, *fimF*, *fimG*, *fimH*, *fimI*) was detected using RT–qPCR. The total volume of the reaction system was 20 μL with one μg total RNA, and the reverse transcription was conducted using SYBR green PCR master mix. The standard cycling parameters were performed on the ABI StepOne Plus instrument, and each target gene was examined three times. The primers of the target genes were listed in Additional file 1.

### Statistical analyses

Statistical analyses were performed with the GraphPad software package (GraphPad Software, LaJolla, CA, USA). One-way analysis of variance (ANOVA) was used to analyze the adhesion and invasion assay data, and two-way ANOVA was used to analyze the survival assay, and RT–qPCR results. The animal infection data were analyzed with the nonparametric Mann–Whitney *U-*test. Each experiment was performed three times and statistical significance indicated as follows: *P < 0.05, **P < 0.01, ***P < 0.001.

## Results

### Identification of strains APEC40-Δ*yqeH* and APEC40-CΔ*yqeH*

**Figure 1:**
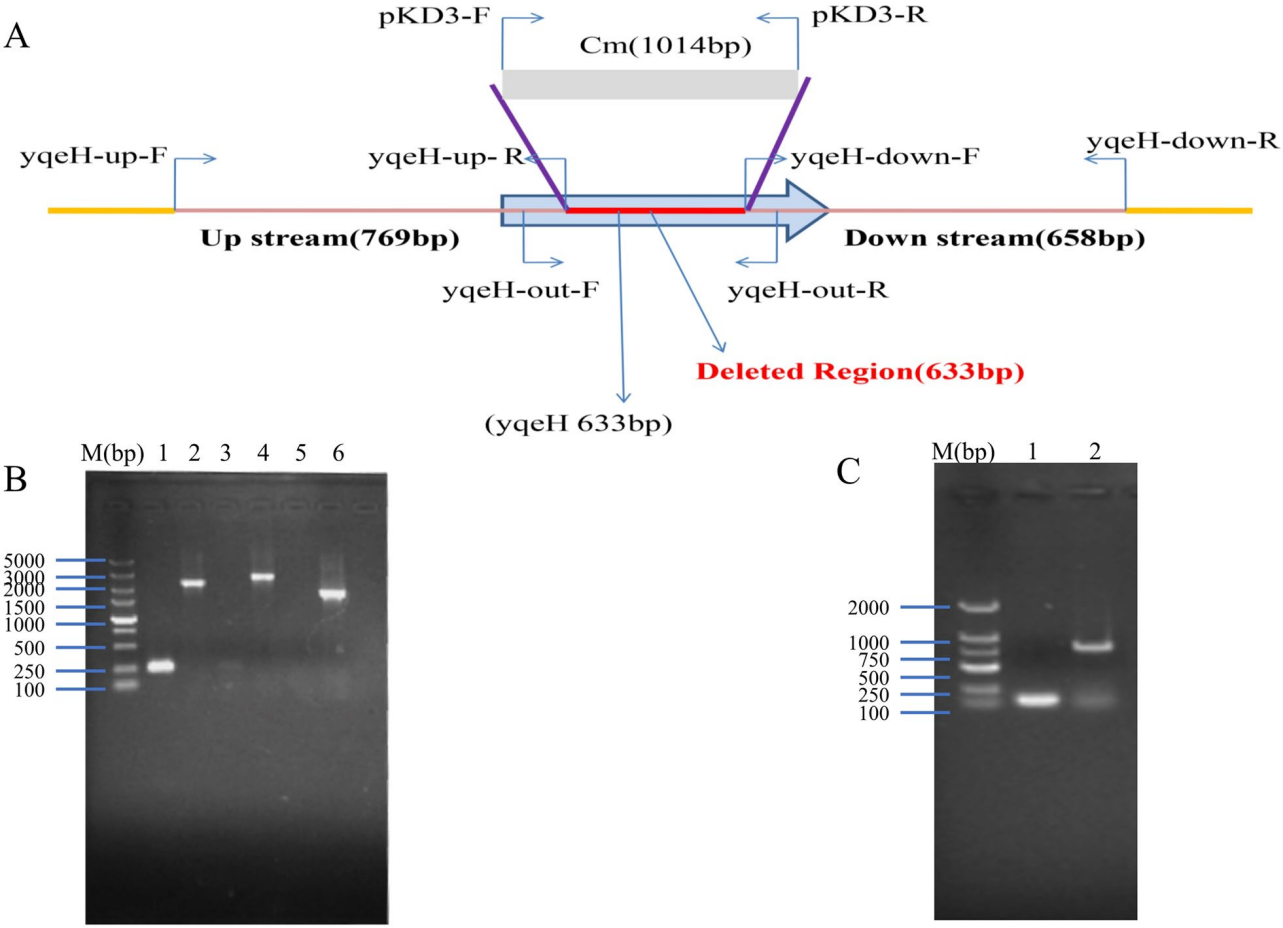
**PCR analysis of mutant strain APEC40-Δ*yqeH* and complemented strain APEC40-CΔ*yqeH*.** **A** Schematic diagram of the strategy used to construct the APEC40 *yqeH* deletion mutant. The *yqeH* gene was deleted by replacing the partial gene sequence with a chloramphenicol-resistance cassette. The primers used for confirmation of the *yqeH* deletion are indicated. **B** Confirmation of the *yqeH* mutant strain APEC40-Δ*yqeH*. Lane identities in (**B**)—M: 2000-bp DNA marker; 1–2: identification of wild-type APEC40-*yqeH* strain (lane 1, 252 bp; lane 2, 2256 bp); 3–4: identification of APEC40-Δ*yqeH*-Cm (lane 3, 0 bp; lane 4, 2637 bp); 5–6: identification of APEC40-Δ*yqeH* (lane 5, 0 bp; lane 6, 1623 bp). **C** Confirmation of the complemented strain APEC40-CΔ*yqeH*. Lane identities in (**C**)—M: 2000-bp DNA marker; 1: Recombinant plasmid pSTV28 blank control identification, 193 bp; 2: APEC40-CΔ*yqeH* identification, 826 bp.

The Δ*yqeH* and CΔ*yqeH* mutant strains were constructed from parental strain APEC40 with the lambda Red recombination method (Figure 1A), and were confirmed with PCR. Using primers *yqeH*-out-F/*yqeH*-out-R, a 2256-bp product was amplified from strain APEC40, whereas a 1623-bp product was amplified from Δ*yqeH* (Figure 1B). The nucleotide sequence of *yqeH* in recombinant plasmid pSTV28-*yqeH* was sequenced, and showed 100% nucleotide sequence identity to the *yqeH* gene of APEC40. The complementary strain APEC40-CΔ*yqeH* was also confirmed by PCR (Figure 1C). A 826 bp PCR product was amplified from APEC40-CΔ*yqeH* with PCR using primers M13F/M13R.

### Characterization of APEC40-Δ*yqeH* mutant strain

**Figure 2:**
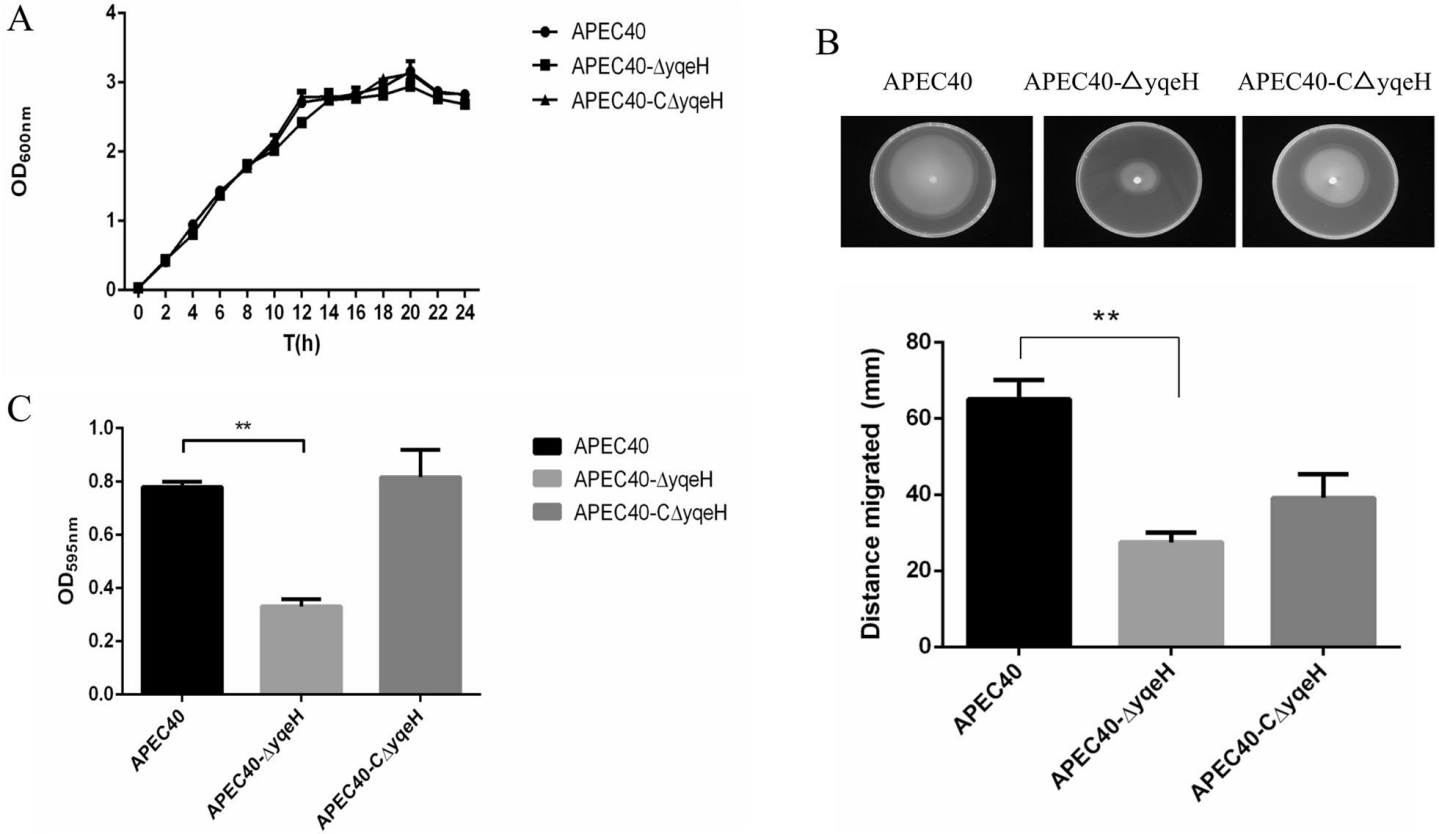
**Characteristics of strains APEC40, APEC40-Δ*yqeH*, and APEC40-CΔ*yqeH*.** **A** Growth curves of APEC40, APEC40-Δ*yqeH*, and APEC40-CΔ*yqeH* in LB broth. Growth rates of APEC40, APEC40-Δ*yqeH*, and APEC40-CΔ*yqeH* were monitored by measuring their OD_600_ values in LB medium at 2 h intervals for 24 h. During culture for 24 h, there was no significant difference in the growth rates of APEC40, APEC40-Δ*yqeH*, and APEC40-CΔ*yqeH* grown in LB broth (*P* > 0.05). Each value is the average of three independent experiments. **B** Bacterial motility. Swimming motility of strain APEC40-Δ*yqeH* was significantly lower than that of the wild-type APEC40 strain, whereas the swimming motility of complemented strain APEC40-CΔ*yqeH* was restored. The experiment was repeated three times and all samples were measured in triplicate. **C** Biofilm formation by APEC strains as determined with crystal violet (CV) staining. APEC40-Δ*yqeH* showed significantly lower biofilm formation than wild-type strain APEC40 after crystal violet staining (**P < 0.01 for the WT vs. mutant).

To determine whether the deletion of *yqeH* influenced the growth of APEC40, bacterial growth was evaluated by measuring OD_600_ from 0 to 24 h. After the deletion of the *yqeH* gene, APEC40-Δ*yqeH* showed a growth rate like that of WT APEC40 (Figure 2A). These data suggest that the deletion of the *yqeH* gene had no significant effect on the growth of APEC40.

The bacterial motility assay showed that APEC40-Δ*yqeH* mutant strain motility was significantly lower than that of the WT strain APEC40 (*P* < 0.05) (Figure 2B). The influence of *yqeH* on the bacterial morphology was examined by transmission electron microscopy. The results revealed remarkable differences in the flagella between the APEC40 and APEC40-Δ*yqeH* strains. Many long flagella were distributed at APEC40 periphery, but APEC40-Δ*yqeH* flagella were few and impaired. A few broken flagella appeared on the surface of APEC40-CΔ*yqeH* (Additional file 2). Moreover, APEC40-Δ*yqeH* formed significantly less biofilm than WT strain APEC40 (Figure 2C).

### Deletion of *yqeH* reduces bacterial adherence and invasion

**Figure 3:**
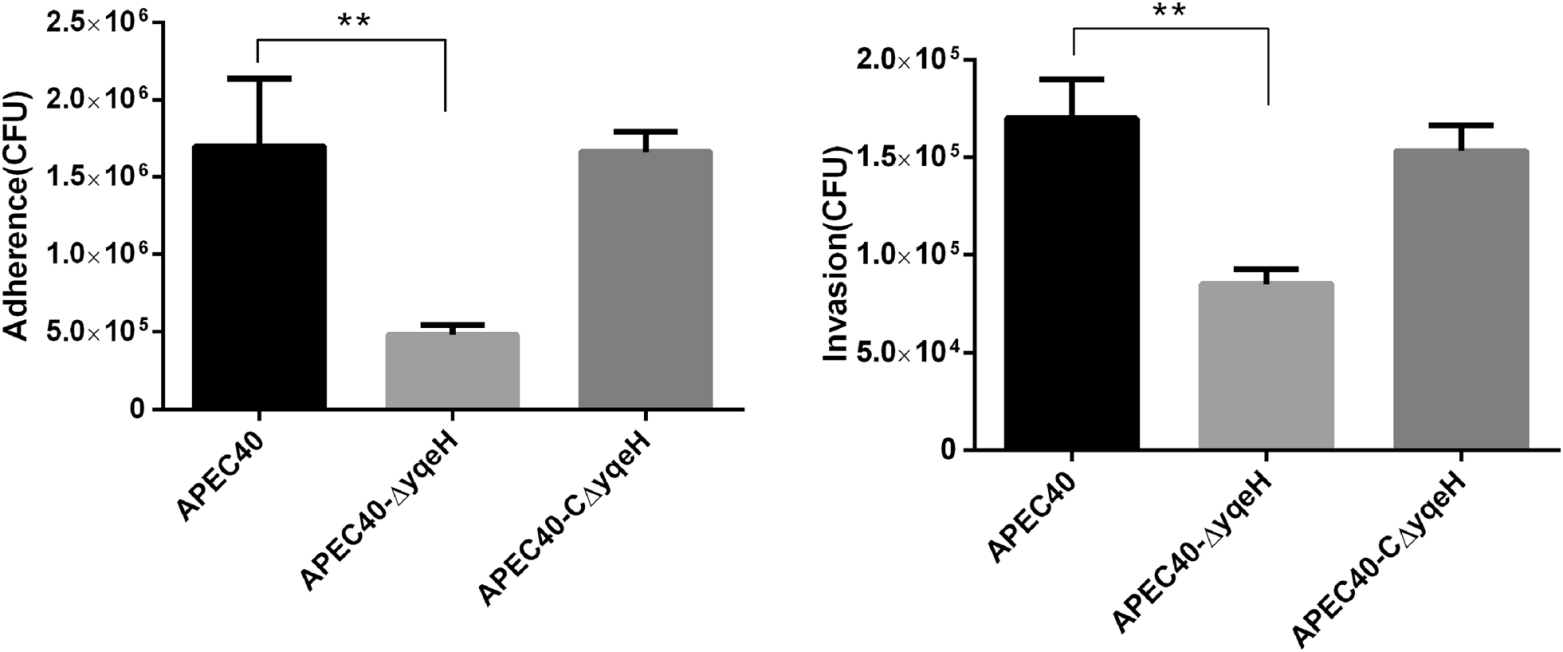
**Deletion of *yqeH* reduced the adherence and invasion abilities of APEC.** Adherence to and invasion of DF-1 cells was significantly lower in mutant strain APEC40-Δ*yqeH* than in WT strain APEC40. (**P < 0.01 for WT vs. mutant).

As shown in Figure 3, the APEC40-Δ*yqeH* mutant showed reduced adherence to and invasion of DF-1 cells compared with those of WT strain APEC40.

### Virulence of mutant strain APEC40-Δ*yqeH*

**Figure 4:**
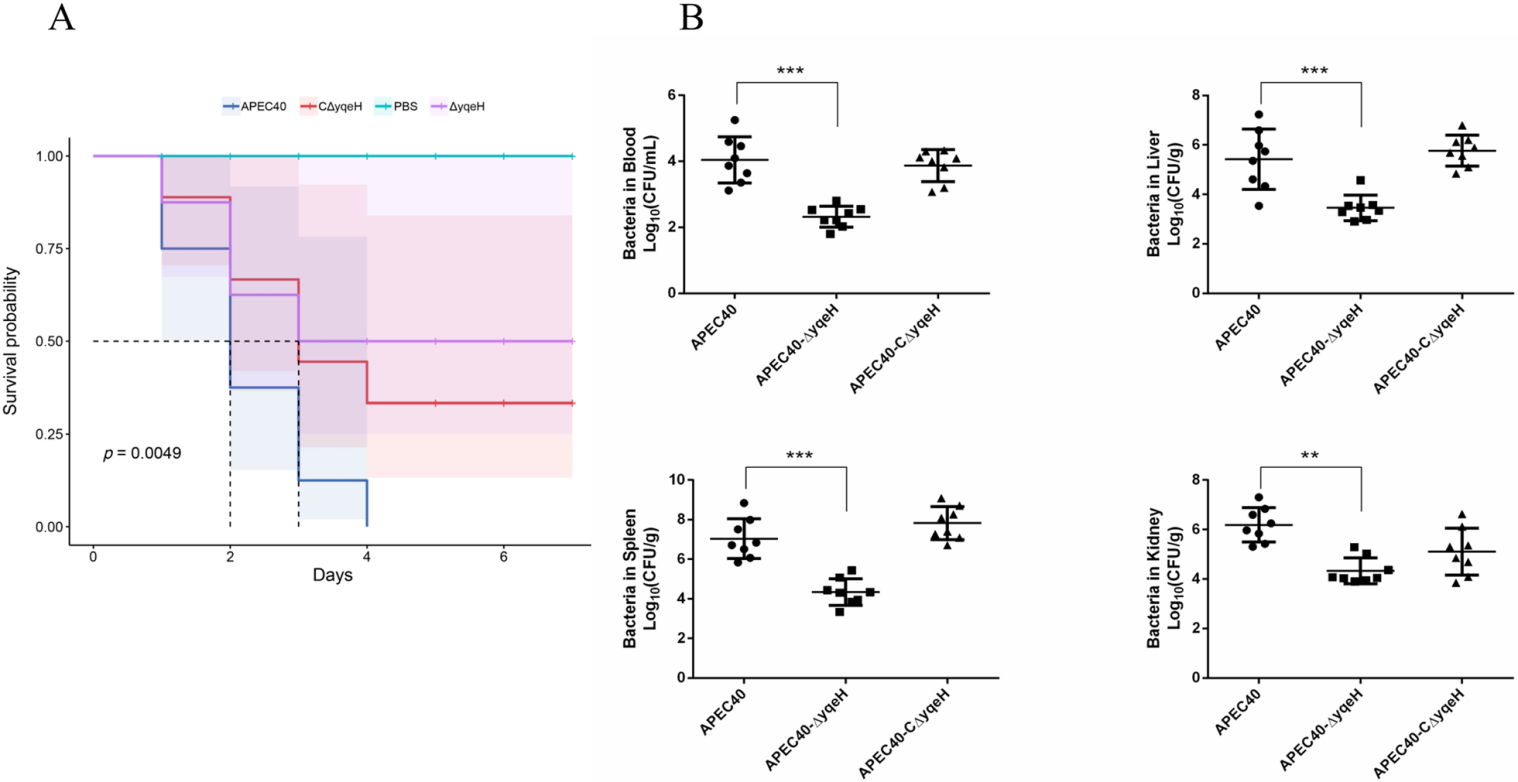
**Animal systemic infection in vivo.** **A** Determination of bacterial virulence. Seven-day-old chicks were inoculated intratracheally with 10^8^ colony-forming units (CFUs) of APEC40, APEC40-Δ*yqeH*, or APEC40-CΔ*yqeH*. Chicks that were administered PBS were used as negative control. Survival was monitored for 7 days post-infection. **B** Bacterial colonization and proliferation in chicks. Groups of eight 7-day-old chicks were intratracheally infected with bacteria (10^8^ CFUs). Bacteria were recovered from the lung, blood, liver, and spleen at 24 h post-infection. The nonparametric Mann–Whitney *U*-test was used to determine statistical significance (**P < 0.01, ***P < 0.001 for WT vs. mutant).

To investigate the effects of YqeH on bacterial pathogenicity, the virulence of the APEC40, APEC40-Δ*yqeH*, and APEC40-CΔ*yqeH* strains was compared in a chick model. As shown in Figure 4A, the mortality rate was significantly lower after infection with strain APEC40-Δ*yqeH* (50%, 4/8) than after infection with strain APEC40 (100%, 8/8). The mortality rate after infection with strain APEC40-CΔ*yqeH* was restored to 75% (6/8) of the WT level. These results indicate that YqeH contributes to APEC virulence.

To measure the influence of YqeH on APEC bacterial numbers in blood and tissues or organs in vivo, a systemic infection experiment was performed to assess bacterial proliferation in chicks. The bacterial loads in the blood, livers, spleens, and kidneys of infected chicks were determined at 24 h post-infection. With the removal of *yqeH*, the bacterial loads of APEC40-Δ*yqeH* were significantly lower than those of WT strain APEC40 in all samples. Moreover, the virulence of APEC40-CΔ*yqeH* was restored (Figure 4B).

### Effects of YqeH on the transcriptional profile of virulence-related genes

**Figure 5:**
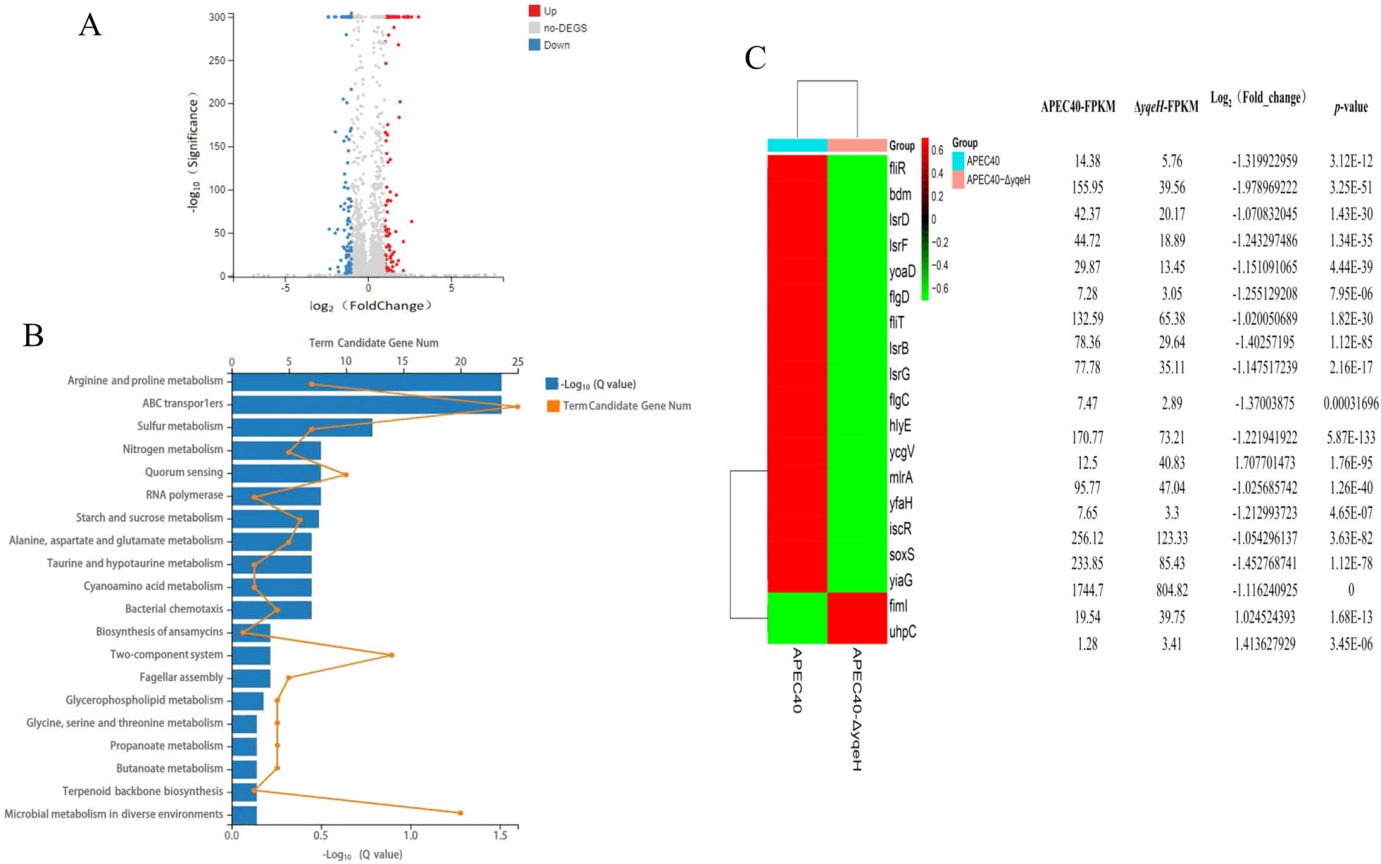
**Analysis of differentially expressed genes by RNA-seq.** **A** Volcano plots were used to visualize the differentially expressed genes in APEC40 and APEC40-Δ*yqeH*. Genes with significantly upregulated expression in APEC40-Δ*yqeH* relative to their expression in APEC40 are indicated with red dots; those downregulated are indicated with blue dots; and grey dots represent genes with no significant difference in expression between the strains. The results showed that 112 genes were upregulated and 101 genes were downregulated in APEC40-Δ*yqeH* relative to their expression in wild-type strain APEC40 (differentially expressed genes were selected at fold change > 2 and q < 0.005). **B** KEGG enrichment analysis of differentially expressed genes. Top 20 pathways enriched in differentially expressed genes are shown in the figure. **C** Quantitative differences in the expression levels of 19 differentially expressed virulence-related genes between wild-type strain APEC40 and mutant strain APEC40-Δ*yqeH* are shown with a heatmap.

A transcriptional analysis showed that 112 genes were upregulated and 101 genes were downregulated in APEC40-Δ*yqeH* relative to their expression in WT strain APEC40 (Figure 5A). The up-and downregulated genes (Additional file 3) were analyzed for their enrichment in KEGG pathways. The differentially expressed genes were enriched in pathways functions related to ABC transport, quorum sensing, bacterial chemotaxis, two-component system and flagellar assembly (Figure 5B). We selected several differentially expressed genes related to biofilm formation, bacterial mobility, and virulence for further study, as shown in Figure 5C.

### Verification of differentially expressed genes with RT–qPCR

**Figure 6:**
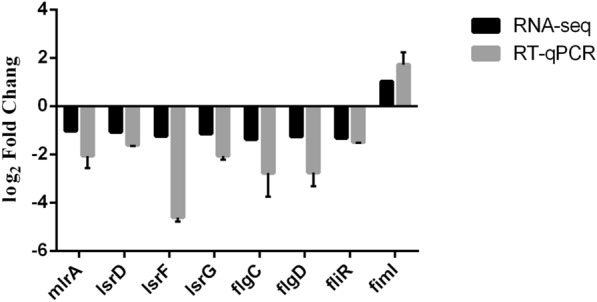
**Verification of RNA-seq results with reverse transcription–quantitative real-time PCR (RT–qPCR).** RT–qPCR was used to determine the expression profiles of several genes differentially expressed in the *yqeH* mutant strain. The x-axis shows the annotations of the selected genes. The *dnaE* gene was used for standardization.

The RNA-seq results were validated with RT–qPCR. Eight genes representing a wide range of gene expression ratios in the *yqeH* mutant were selected for a comparative RT–qPCR analysis. The gene expression patterns determined with RT–qPCR were highly concordant with the RNA-seq results (Figure 6).

### Expression variations of type 1 fimbriae genes

The expression of type 1 fimbriae genes *fimA*, *fimB*, *fimC*, *fimD*, *fimE*, *fimF*, *fimG*, *fimH* and *fimI* showed no significant difference between the wild-type strain APEC40 and the *yqeH* mutant strain (Additional file 4).

## Discussion

The ETT2 locus is present in most *E. coli* strains. However, the transcriptional regulators located in the ETT2 locus are considered key factors in the pathogenicity of *E. coli* [20]. Genomic studies have identified several transcriptional regulators, including YqeK/EtrB, EtrA, and EivF, located at the ETT2 locus, which regulate the expression of virulence genes in pathogenic *E. coli* [8]. In this report, we propose for the first time that YqeH is a transcriptional regulator located at the ETT2 locus of APEC, and describe it. We constructed a YqeH deletion mutant to identify the role of YqeH in APEC pathogenesis. However, the rescue assays for expression of YqeH in YqeH-mutant strain resulted in partial recovery. It is possibly due to deficient expression of YqeH by using a low-copy plasmid, pSTV28, as the complementation plasmid.

Biofilm formation is an important process by which bacteria establish infections, leading to host diseases [21]. In this study, APEC biofilm formation was significantly reduced after *yqeH* deletion compared with that of WT strain APEC40. Biofilm formation is a complex process, during which the expression of specific genes is required to produce specific substances that promote and regulate biofilm formation [22]. The effects of YqeH on the expression of biofilm-related genes in APEC were investigated by using a transcriptional analysis. In total, seven downregulated genes related to biofilm formation were enriched in differentially expressed genes of YqeH-deletion mutant compared with WT strain. Among them, *mlrA*, *yoaD* and *bdM* were reported for their role in c-di-GMP signaling pathway. Moreover, it was reported that *lsrB*, *lsrD*, *lsrF* and *lsrG* mediated the biosynthesis of AI-2 protein in the quorum sensing signaling pathway. As a signaling molecule, AI-2 promotes the transformation of bacteria from suspension to biofilm modes, and promotes the transcription of bacterial genes that facilitate biofilm formation [23]. The second messenger c-di-GMP regulates biofilm formation by changing the concentrations of bacteria within host cells [24]. Our results show that after the deletion of *yqeH*, the expression levels of genes related to the synthesis of AI-2 and c-di-GMP were lower than in WT strain APEC40, which ultimately affected the formation of the APEC biofilm.

The flagellum is a motor organelle and a protein export apparatus that controls bacterial motility and behavior [25]. In this study, bacterial motility was significantly reduced after *yqeH* deletion compared with that in WT strain APEC40. Transcriptomic profiling showed that the transcription of *flgC*, *flgD*, *fliT*, and *fliR* was similarly reduced. FlgC is the flagellar basal-body protein; FlgD is essential for flagellar hook formation; FliT is the flagellar synthesis and assembly chaperone protein; and FliR is the flagellar export pore protein [26, 27]. Therefore, we speculate that the reduced expression of these genes impaired flagellum synthesis in the bacteria, resulting in reduced bacterial motility.

The bacterial colonization and invasion of host cells are crucial steps in APEC infection process [28, 29]. Type 1 fimbriae contribute to bacterial adhesion and invasion of host cells, which create conditions for a series of processes, such as pathogen colonization and infection [30, 31]. However, our results (Additional file 4) showed that the transcript expression of type 1 fimbriae genes had no significant differential changes after the deletion of *yqeH*. The flagellum has been shown to mediate bacterial adhesion and invasion, and is implicated in the virulence of pathogenic bacteria. The motility of the flagellum is an important virulence feature of many bacterial pathogens and is necessary for the establishment of infection [32]. For example, the FlgC protein plays an important role in the binding of *Salmonella enteritidis* to host epithelial cells [33], and the deletion of the flagellin *fliR* gene affects the adhesion and pathogenicity of bacteria to host cells [34]. Biofilm formation is also strongly related to the ability of bacteria to adhere to and invade cells. Several studies have shown that the biofilm-synthesis-related gene *pgaC*, encoding N-glycosyltransferase, not only mediates biofilm formation, but also plays an important role in bacterial adhesion to its host cell [35]. The quorum-sensing transcriptional activator SdiA regulates the expression of the *rck* gene, which mediates *E. coli* adhesion to and invasion of epithelial cells [36]. Our results show that the loss of *yqeH* reduced bacterial motility and biofilm formation, which may have accounted for the diminished adhesion to and invasion of DF-1 cells by mutant APEC40-Δ*yqeH*. The adhesion and colonization abilities of APEC in the host blood and tissues are key factors in its pathogenesis [37]. The numbers of viable cells of deleted strain APEC40-Δ*yqeH* in the host blood and various tissues were significantly lower than those in the WT strain. Our experimental results demonstrate that YqeH is involved in APEC virulence.

In summary, we have demonstrated for the first time that the transcriptional regulator YqeH is involved in the motility, biofilm formation, and virulence of APEC strains. This study provides a basis for further functional research into the pathogenic role of YqeH in APEC.

## Supplementary Information

**Additional file 1**: **Primers used in this study**.

**Additional file 2**: **Bacterial micromorphology of APEC40, APEC40-ΔyqeH and APEC40-CΔyqeH observed by transmission electron microscopy**.

**Additional file 3**: **Differentially expressed genes data**.

**Additional file 4**: **Expressions of type 1 fimbriae genes of the wild-type strain APEC40, mutant strain APEC40-ΔyqeH and complementary strain APEC40-CΔyqeH were tested by RT-qPCR**.

## Data Availability

The sequence was deposited in the GenBank database (Accession number PRJNA805014).
