# Supplementary material for: YqeH contributes to avian pathogenic Escherichia coli pathogenicity by regulating motility, biofilm formation, and virulence
Source: Vet Res. 2022 Apr 18;53:30. doi: 10.1186/s13567-022-01049-6 (PMC9014576; doi:10.1186/s13567-022-01049-6)
Supplement: Supplementary file 1 — Additional file 1: Primers used in this study. [file 13567_2022_1049_MOESM1_ESM.docx]

**Additional file 1. Primers used in this study**

| **Primer** | **Sequence (5’to 3’)** |
| --- | --- |
| *yqeH-*in*-F* | CCAAATAATGCCAGAAGA |
| *yqeH-*in*-R* | TTGCGGTCGCTGACTAAA |
| *yqeH*-out-F | TTTCTATTCCACCTTCGT |
| y*qeH*-out-R | GTGCGTCTGTTGATGAGT |
| *yqeH*-up-F  y*qeH*-lap-cm-up-R | CAAAACAACACTACAGCAGGTT  CCAGCCTACACGACTACAAAGCCCCATG |
| *yqeH*-lap-cm-down-F | TATTCATATGGGTCCAACATTGATACTC |
| *yqeH*-down-R | AATCCTCTTCTCGGCACT |
| pkd3-cm-lap-*yqeH*-F | GGAGGTTCTTAACATGGGGCTTTGTAGTCGTGTAGGCTGGAGCTGCTT |
| pkd3-cm-lap-*yqeH*-R | TGGCAGATTGTTGAGTATCAATGTTGGACCCATATGAATATCCTCCTTAGTTC |
| *yqeH* Co-F | CCCAAGCTTTTTTCATAATGTGCATTTGT |
| *yqeH* Co-R | CGCGGATCCTGGCAGATTGTTGAGTATCAA |
| *M13*-F | CAGGAAACAGCTATGAC |
| *M13*-R | GTTTTCCCAGTCACGAC |
| *mlrA*-F | ATAGCCTGCGAACGTGGAT |
| *mlrA*-R | GTCGGTTGTTGGTACTGAAGC |
| *lsrD*-F | GGCGATTCTCCTGACCTT |
| *lsrD*-R | CAAACAGATACAGCGTGCC |
| *lsrF*-F | ACTGGCGTGGGCAAAGAC |
| *lsrF*-R | TCCGGCAACAATCCGTTC |
| *lsrG*-F | TTTTCGCCAGAACCACCT |
| *lsrG-R* | CCACTGCGTCTTCATCTTT |
| *flgC*-F | CGAATGCTGATAGCGTGAC |
| *flgC*-R | ATAACATCGGCAACCTTTAC |
| *flgD*-F | CTGACTTTGTTGGTGGCG |
| *flgD*-F | CCGAGCGTGGTATTGAGTT |
| *fliR*-F | CAGGTGACAAGCGAACAA |
| *fliR*-R | ATTGCCAGACCCAGTTTT |
| *fimA*-F | ACAGGACGGAGCAACCAG |
| *fimA*-R | TGACCCGCATCAATCACC |
| *fimB*-F | CCGCTATTGAACAAAGAA |
| *fimB*-R | AATCTCCAGTGACAACCC |
| *fimC*-F | ATCGTGACGCCTCCTCTG |
| *fimC*-R | AACTTTCCCGGTCCTGTG |
| *fimD*-F | GCCGAAATTCACCGACTA |
| *fimD*-R | GATGGCTACCACTCAAATACAG |
| *fimE*-F | CGGTAAAGAAGTTCAGGC |
| *fimE*-R | GTTCACTAATACGCATCCC |
| *fimF*-F | TACCGGCGTTGCAGATAG |
| *fimF*-R | CGGACGATGGAGCATTAA |
| *fimG*-F | TCTCGGCGATCTTTATTCT |
| *fimG*-R | AGTGACCCTTGACGTTCC |
| *fimH*-F | CTATAACAGCGATGATTTCCA |
| *fimH*-R | GGTGACATCACGAGCAGAA |
| *fimI*-F | GCCAGGGATAGCCACCAA |
| *fimI*-R | CGATGAAATGTAGCGAAGTAGA |
| *dnaE*-F | GATTGAGCGTTATGTCGGAGGC |
| *dnaE*-R | GCCCCGCAGCCGTGAT |
