# Supplementary material for: YqeH contributes to avian pathogenic Escherichia coli pathogenicity by regulating motility, biofilm formation, and virulence
Source: Vet Res. 2022 Apr 18;53:30. doi: 10.1186/s13567-022-01049-6 (PMC9014576; doi:10.1186/s13567-022-01049-6)
Supplement: Supplementary file 2 — Additional file 2: Bacterial micromorphology of APEC40, APEC40-ΔyqeH and APEC40-CΔyqeH observed by transmission electron microscopy. [file 13567_2022_1049_MOESM2_ESM.docx]

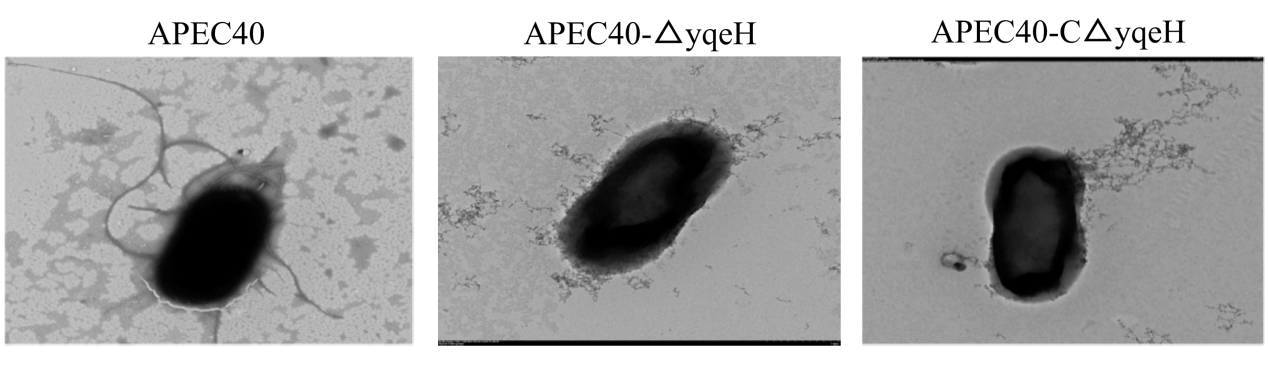


**Additional file 2. Bacterial micromorphology of APEC40, APEC40-Δ*yqeH* and APEC40-CΔ*yqeH* observed by transmission electron microscopy.**
