# Supplementary material for: YqeH contributes to avian pathogenic Escherichia coli pathogenicity by regulating motility, biofilm formation, and virulence
Source: Vet Res. 2022 Apr 18;53:30. doi: 10.1186/s13567-022-01049-6 (PMC9014576; doi:10.1186/s13567-022-01049-6)
Supplement: Supplementary file 4 — Additional file 4: Expressions of type 1 fimbriae genes of the wild-type strain APEC40, mutant strain APEC40-ΔyqeH and complementary strain APEC40-CΔyqeH were tested by RT-qPCR. [file 13567_2022_1049_MOESM4_ESM.docx]

**Additional file 4.** **Expressions of type 1 fimbriae genes of the wild-type strain APEC40, mutant strain APEC40-Δ*yqeH* and complementary strain APEC40-CΔ*yqeH* were tested by RT-qPCR.**
